# Small Hepatitis B Virus Surface Antigen Promotes Hepatic Gluconeogenesis via Enhancing Glucagon/cAMP/Protein Kinase A/CREB Signaling

**DOI:** 10.1128/jvi.01020-22

**Published:** 2022-11-17

**Authors:** Yan Chen, Biao Wang, Xiaowei Ou, Yidan Wu, Yun He, Xinjian Lin, Xu Lin

**Affiliations:** a Key Laboratory of Gastrointestinal Cancer ( Fujian Medical University), Ministry of Education, Fuzhou, China; b Fujian Key Laboratory of Tumor Microbiology, Department of Medical Microbiology, Fujian Medical University, Fuzhou, China; University of Southern California

**Keywords:** hepatitis B virus, small hepatitis B virus surface antigen, gluconeogenesis, cAMP/PKA/CREB signaling, diabetes mellitus

## Abstract

Hepatitis B virus (HBV) is a major risk factor for serious liver diseases. The liver plays a unique role in controlling carbohydrate metabolism to maintain the glucose level within the normal range. Chronic HBV infection has been reported to associate with a high prevalence of diabetes. However, the detailed molecular mechanism underlying the potential association remains largely unknown. Here, we report that liver-targeted delivery of small HBV surface antigen (SHBs), the most abundant viral protein of HBV, could elevate blood glucose levels and impair glucose and insulin tolerance in mice by promoting hepatic gluconeogenesis. Hepatocytes with SHB expression also exhibited increased glucose production and expression of gluconeogenic genes *glucose-6-phosphatase* (*G6pc*) and *phosphoenolpyruvate carboxykinase* (*PEPCK*) in response to glucagon stimulation. Mechanistically, SHBs increased cellular levels of cyclic AMP (cAMP) and consequently activated protein kinase A (PKA) and its downstream effector cAMP-responsive element binding protein (CREB). SHBs-induced activation of CREB enhanced transcripts of gluconeogenic genes, thus promoting hepatic gluconeogenesis. The elevated cAMP level resulted from increased transcription activity and expression of adenylyl cyclase 1 (AC1) by SHBs through a binary E-box factor binding site (BEF). Taken together, we unveiled a novel pathogenic role and mechanism of SHBs in hepatic gluconeogenesis, and these results might highlight a potential target for preventive and therapeutic intervention in the development and progression of HBV-associated diabetes.

**IMPORTANCE** Chronic HBV infection causes progressive liver damage and is found to be a risk factor for diabetes. However, the mechanism in the regulation of glucose metabolism by HBV remains to be established. In the current study, we demonstrate for the first time that the small hepatitis B virus surface antigen (SHBs) of HBV elevates AC1 transcription and expression to activate cAMP/PKA/CREB signaling and subsequently induces the expression of gluconeogenic genes and promotes hepatic gluconeogenesis both *in vivo* and *in vitro*. This study provides a direct link between HBV infection and diabetes and implicates that SHBs may represent a potential target for the treatment of HBV-induced metabolic disorders.

## INTRODUCTION

Hepatitis B virus (HBV) infection is a serious public health problem, with 250 million people infected chronically worldwide (1). Given the pivotal role of the liver in glucose homeostasis and the fact that liver diseases of various etiologies may contribute to diabetes, the association between HBV infection and glucose metabolism abnormality has attracted considerable attention. Patients with chronic HBV infection have a high prevalence of impaired fasting glucose and glucose intolerance (2, 3). Several studies have suggested that there is a strong association between HBV infection and diabetes (4–6). Successful vaccination against HBV has had an obvious protective effect on diabetes (7). Thus, HBV infection has been increasingly recognized as an important risk factor for impaired fasting glucose, glucose intolerance, and the subsequent development of diabetes. However, the exact underlying mechanism involved in the regulation of glucose metabolism by HBV is still unclear.

Hyperglycemia and glucose intolerance in diabetes are caused largely by dysregulated glucose production in the liver (8). Excessive gluconeogenesis rather than glycogenolysis contributes primarily to aberrant hepatic glucose production in type 2 diabetes (9). Gluconeogenesis is determined by a balance between glucagon and insulin (10). Under the fasting state, glucagon is released from islet alpha cells and initiates hepatic gluconeogenesis through a specific glucagon receptor (GCGR). Adenylyl cyclase (AC) is then activated to increase cellular levels of cyclic AMP (cAMP) that can regulate the transcription of various target genes mainly through protein kinase A (PKA) and its downstream effectors, such as cAMP response element binding protein (CREB). In addition to insulin, glucagon also plays a key pathogenic role in the development of diabetes (11). Plasma glucagon levels in diabetics are inappropriately elevated, which contributes to increased hepatic glucose production and hyperglycemia (12). Deletion of GCGR maintained normal blood glucose and improved glucose tolerance in diabetes (13, 14). Therefore, hepatic glucagon signaling has now become an attractive target for diabetic therapy.

Small hepatitis B virus surface antigen (SHBs), the major envelop protein of HBV, is encoded by the PreS/S open reading frame. SHBs is highly expressed which far exceeds the requirement of HBV virion assembly and forms subviral particles. Excess production of these subviral particles may be immunological decoys for HBV-specific humoral immunity to promote a state of virus-specific T cell anergy and deletion (15). In addition to adaptive immunity, SHBs has also been shown to suppress the innate immunity (16). Moreover, our prior study has found that SHBs enhanced Fas-mediated hepatic apoptosis and increased the susceptibility of mice to acute liver failure (17). HBsAg seroclearance is considered a functional cure (18). Thus, SHBs has been identified to be an important pathogenic factor of HBV. A proteomic analysis has demonstrated that 45% of differentially expressed proteins in the liver of HBsAg-positive mice compared with those of the HBsAg-negative mice were involved in lipid, carbohydrate, and amino acid metabolism (19). It raised the possibility of SHBs in the regulation of hepatic metabolism.

However, to the best of our knowledge, whether SHBs regulates hepatic glucose metabolism has yet to be elucidated. In this study, we aimed to assess the impact of SHBs on hepatic gluconeogenesis and to explore the possible underlying mechanism. We found that SHBs expression induces hyperglycemia and glucose intolerance by enhancing glucagon-stimulated hepatic gluconeogenesis.

## RESULTS

### SHBs enhances glucose production and gluconeogenesis in primary hepatocytes.

**FIG 1:**
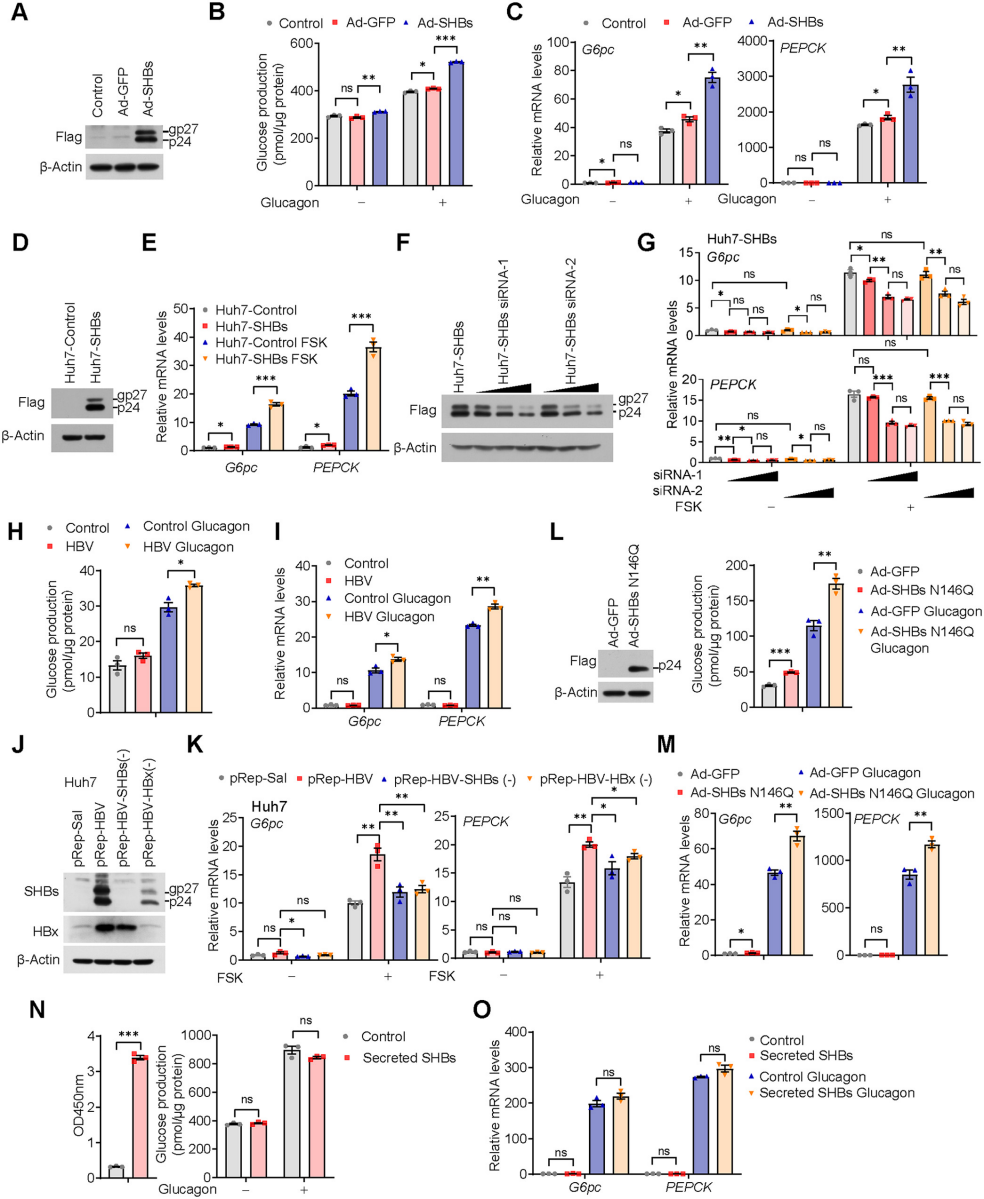
SHBs regulates gluconeogenesis in hepatocytes. (A) Expression of SHBs in Ad-SHBs-infected mouse primary hepatocytes confirmed by Western blot analysis. Mouse primary hepatocytes were untreated or infected with Ad-GFP or Ad-SHBs for 48 h. (B) Effect of SHBs on glucose production in mouse primary hepatocytes. At 36 h after infection with Ad-SHBs, the mouse primary hepatocytes were serum starved overnight and subjected to a glucose production assay. Hepatic glucose production was measured 6 h after glucagon (100 nM) stimulation and normalized by protein levels. (C) Relative mRNA expression levels of *G6pc* and *PEPCK* in untreated or Ad-GFP- and Ad-SHBs-infected mouse primary hepatocytes exposed to glucagon (100 nM) for 2 h as determined by RT-qPCR. (D) Expression of SHBs in Huh7-SHBs cells confirmed by Western blot analysis. (E) Relative mRNA expression levels of *G6pc* and *PEPCK* in Huh7-Control and Huh7-SHBs cells exposed to FSK (10 μM) for 2 h as determined by RT-qPCR. (F) siRNA-mediated knockdown of SHBs in Huh7-SHBs cells confirmed by Western blot analysis. (G) Effect of SHBs knockdown on the mRNA expression levels of *G6pc* and *PEPCK* in Huh7-SHBs cells determined by RT-qPCR. (H) Effect of HBV infection on glucose production in primary human hepatocytes (PHHs). PHHs were infected with HBV virions at MOI of 1,000 VGE in the presence of 2% DMSO and 4% PEG 8000. At 7 days after infection, cells were subjected to a glucose production assay. Hepatic glucose production was measured 6 h after glucagon (100 nM) stimulation and normalized by protein levels. (I) Relative mRNA expression levels of *G6pc* and *PEPCK* in HBV-infected PHHs exposed to glucagon (100 nM) for 2 h as determined by RT-qPCR. (J) Expression of SHBs and HBx in pRep-HBV, pRep-HBV-SHBs (−), pRep-HBV-HBx (−), or control plasmid pRep-Sal I transfected Huh7 cells determined by Western blot analysis. (K) Relative mRNA expression levels of *G6pc* and *PEPCK* in pRep-HBV, pRep-HBV-SHBs (−), pRep-HBV-HBx (−), or control plasmid pRep-Sal I transfected Huh7 cells exposed to FSK (10 μM) for 2 h. (L) Effect of SHBs N146Q on glucose production in mouse primary hepatocytes. Cells were infected with Ad-SHBs N146Q and then subjected to a glucose production assay (right). The expression of SHBs N146Q was confirmed by Western blot analysis (left). (M) Relative mRNA expression levels of *G6pc* and *PEPCK* in Ad-SHBs N146Q-infected mouse primary hepatocytes exposed to glucagon (100 nM) for 2 h as determined by RT-qPCR. (N) Effect of secreted SHBs on glucose production in mouse primary hepatocytes. Secreted SHBs after concentration was detected by ELISA (left). Cells were treated with secreted SHBs for 48 h and then subjected to glucose production assay (right). (O) *G6pc* and *PEPCK* mRNA expression levels in secreted SHBs-treated mouse primary hepatocytes detected by RT-qPCR. The glycosylated (gp) and nonglycosylated (p) forms of SHBs were indicated. Data are presented as means ± SEM. *, *P < *0.05; **, *P < *0.01; ***, *P < *0.001; ns, no significant difference.

To investigate the effect of SHBs on hepatic gluconeogenesis at the cellular level, we utilized mouse primary hepatocytes isolated from C56BL/6 mice and then infected them with adenoviruses expressing SHBs (Ad-SHBs). After confirming SHBs was expressed in the mouse primary hepatocytes (Fig. 1A), we performed a glucose production assay in those cells treated with glucagon. The results showed that SHBs significantly increased glucose production in the mouse primary hepatocytes compared with Ad-GFP infection (Fig. 1B). Consistently, mRNA expression of *G6pc* or *PEPCK*, the two rate-limiting enzymes for gluconeogenesis, was significantly increased by SHBs under glucagon treatment (Fig. 1C). Notably, untreated/uninfected controls and an unrelated protein overexpression control of Ad-GFP relative to Ad-SHBs were included to exclude the nonspecific effect of SHBs expression. As another measure of assurance on the effect of SHBs on hepatic gluconeogenesis, an SHBs stably expressed Huh7 cell line (Huh7-SHBs) was established (Fig. 1D) and then treated with forskolin (FSK), the agonist of adenylate cyclase. Under the same stimulation of FSK, Huh7-SHBs cells had a higher mRNA expression of the two gluconeogenic genes than the control cells (Huh7-Control) (Fig. 1E). In contrast, small interfering RNA (siRNA)-mediated knockdown of SHBs in Huh7-SHBs cells significantly diminished *G6pc* or *PEPCK* expression in a dose-dependent manner (Fig. 1F and G). To assess the effect of SHBs in a natural HBV infection system, primary human hepatocytes (PHHs) were infected with HBV virions and treated with glucagon. As shown in Fig. 1H and I, HBV infection in PHHs significantly increased glucose production and gluconeogenic gene expression. Additionally, a 1.2-unit-length HBV genome (pRep-HBV), SHBs-deficient mutant pRep-HBV-SHBs (−), and hepatitis B virus X protein (HBx)-deficient mutant pRep-HBV-HBx (−) were also employed. As expected, there was no SHBs or HBx expression in pRep-HBV-SHBs (−) or pRep-HBV-HBx (−) transfected cells (Fig. 1J). The mRNA levels of *G6pc* and *PEPCK* were elevated in Huh7 cells transfected with pRep-HBV under FSK treatment. However, the SHBs-deficient mutant pRep-HBV-SHBs (−) or HBx-deficient mutant pRep-HBV-HBx (−) attenuated the enhancement (Fig. 1K). As HBx has been reported to promote gluconeogenic gene expression (20), the HBx deficient mutant was used as a parallel control. Intriguingly, depletion of SHBs did not affect HBx expression, whereas HBx deficiency dramatically decreased SHB expression (Fig. 1J), which might suggest that SHBs should play a major role in the regulation of gluconeogenesis. Taken together, these results suggest that SHBs should be a critical player of HBV that stimulates the expression of the genes responsible for hepatic gluconeogenesis.

SHBs is a glycoprotein with a single N-linked glycosylation site at N146, which is important for HBV virion secretion (21, 22). To assess the role of SHBs glycosylation in the regulation of gluconeogenesis, we constructed the adenoviral vector Ad-SHBs N146Q that contains an N146Q point mutation, and the resulting viruses were used to infect mouse primary hepatocytes. As shown in the Fig. 1L and 1M, the lack of glycosylation in SHBs did not affect its ability to enhance glucose production and gluconeogenic gene expression under glucagon stimulation. Therefore, it appears that SHBs-mediated gluconeogenesis is not related to its glycosylation status. SHBs is produced intracellularly at a high rate and can be secreted out of the cell (23). To clarify whether secreted SHBs might take part in the regulation of gluconeogenesis, the culture medium of Huh7-SHBs cells was collected and used to treat mouse primary hepatocytes. There was no appreciable difference in glucose production and gluconeogenic gene expression between secreted SHBs-treated and control mouse primary hepatocytes (Fig. 1N and O). These data indicate that it is the intracellular SHBs that plays a major part in promoting hepatic gluconeogenesis.

### SHBs overexpression increases hepatic gluconeogenesis *in vivo*.

**FIG 2:**
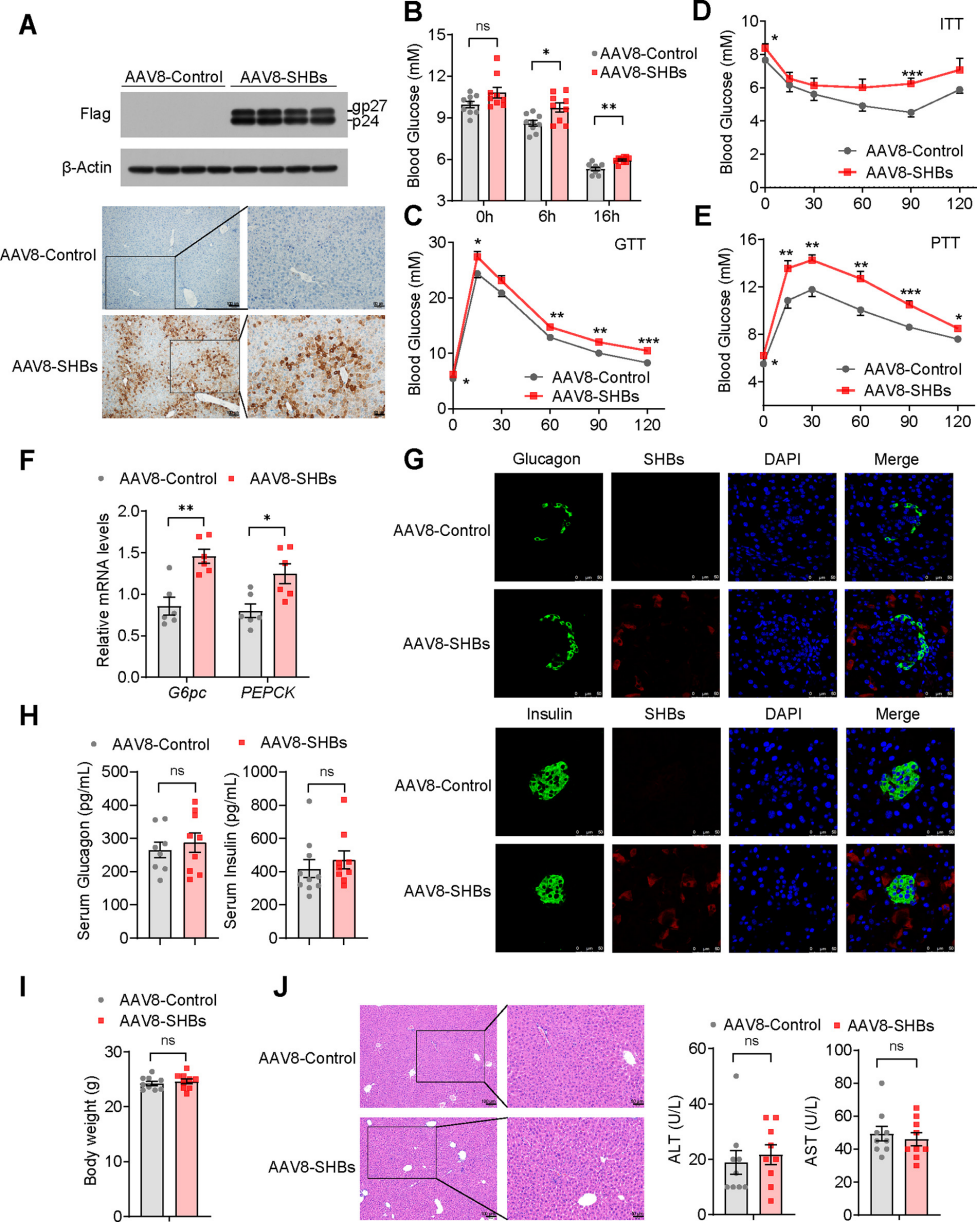
SHBs regulates hepatic gluconeogenesis in mice. (A) Western blot analysis (top) and immunohistochemical analysis (bottom) of SHBs expression in the liver of mice after 3 weeks of AAV8-SHBs injection. The glycosylated (gp) and nonglycosylated (p) forms of SHBs were indicated. (B) Blood glucose levels in AAV8-Control and AAV8-SHBs mice in the fed group and at 6 h and 16 h after fasting (*n* = 9 mice/group). (C to E) AAV8-Control and AAV8-SHBs mice were fasted for 16 h in GTT (C) and PTT (E) or fasted for 5 h in ITT (D). Blood glucose levels were measured at the indicated times after an intraperitoneal injection of 2 g/kg glucose (GTT), 2 g/kg pyruvate (PTT), or 0.5 U/kg insulin (ITT) (*n* = 10 to 12 mice/group). (F) Relative mRNA levels of hepatic gluconeogenic genes in the liver of AAV8-Control and AAV8-SHBs mice (*n* = 6 mice/group). (G) Expression of SHBs in pancreatic islets of AAV8-Control and AAV8-SHBs mice detected by immunofluorescence staining. The sections were immunostained for SHBs (red) and glucagon or insulin (green) as indicated. (H, I) The serum glucagon and insulin levels (H), and body weight (I) of AAV8-Control and AAV8-SHBs mice fasted for 16 h (*n* = 8 to 10 mice/group). (J) Hematoxylin-eosin staining on serial sections of livers (left) and serum ALT and AST analysis (middle and right, *n* = 9 mice/group) were performed between AAV8-Control and AAV8-SHBs mice. Data are presented as means ± SEM. *, *P < *0.05; **, *P < *0.01; ***, *P < *0.001; ns, no significant difference.

To characterize the impact of SHBs in hepatic gluconeogenesis *in vivo*, we overexpressed SHBs in the liver by tail vein injection of the liver-targeted adeno-associated virus 8 (AAV8) into male C56BL/6 mice. Three weeks after injection, a high-level expression of SHBs in the liver was confirmed by Western blot analysis and immunohistochemistry (IHC) (Fig. 2A). The levels of blood glucose were higher than those with AAV8-Control littermates in the fasted state (Fig. 2B). Moreover, AAV8-SHBs mice displayed impaired glucose tolerance and insulin sensitivity (Fig. 2C and D). We speculated that abnormal glucose production derived from dysregulated gluconeogenesis might account for SHBs-induced elevation of glucose levels, glucose intolerance, and decreased insulin sensitivity. To test this hypothesis, a pyruvate tolerance test (PTT) was also performed to examine the effect of SHBs on hepatic gluconeogenesis. As shown in Fig. 2E, the level of blood glucose in AAV8-SHBs mice was higher than that in the control littermates after pyruvate injection. Expression of *G6pc* and *PEPCK* was significantly upregulated in the liver of AAV8-SHBs mice (Fig. 2F). Recombinant AAV8 tail vein injection might cause SHBs expression in pancreatic islets, which could possibly change glucagon and insulin secretion. To exclude this possibility, immunofluorescence staining was performed. In AAV8-SHBs mice, SHBs was shown to express in the pancreas but not in glucagon-producing pancreatic islet alpha cells or insulin-producing pancreatic islet beta cells (Fig. 2G). Serum levels of glucagon or insulin were also unaffected (Fig. 2H). In addition, there was no appreciable difference in body weight, liver histology, or serum levels of alanine transaminase (ALT) and aspartate transaminase (AST) between AAV8-SHBs mice and control littermates (Fig. 2I and J).

**FIG 3:**
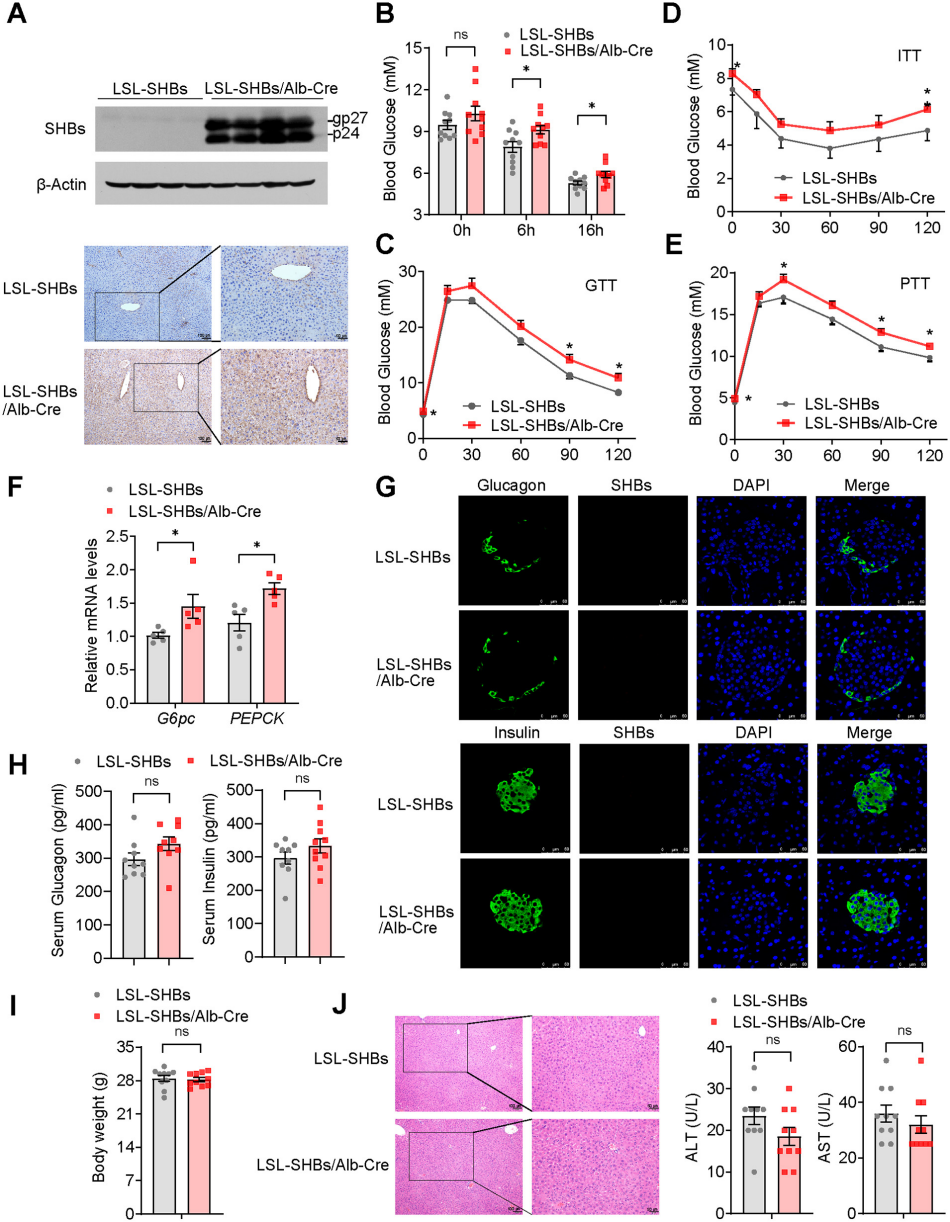
SHBs transgenic mice exhibited a higher gluconeogenesis than control mice. (A) Western blot analysis (top) and immunohistochemical analysis (bottom) of SHBs expression in the liver of LSL-SHBs/Alb-Cre mice. The glycosylated (gp) and nonglycosylated (p) forms of SHBs were indicated. (B) Blood glucose levels of LSL-SHBs and LSL-SHBs/Alb-Cre mice in the fed group and at 6 h and 16 h fasted state (*n *= 10 mice/group). (C to E) Blood glucose levels were measured in LSL-SHBs and LSL-SHBs/Alb-Cre mice at 0, 15, 30, 60, 90, and 120 min after 16 h of fasting and intraperitoneal injection of 2 g/kg glucose (GTT) or pyruvate (PTT) or after 5 h of fasting and intraperitoneal injection of 0.5 U/kg insulin (ITT) (*n* = 6 to 10 mice/group). (F) Relative mRNA expression levels of hepatic gluconeogenic genes in LSL-SHBs and LSL-SHBs/Alb-Cre mice (*n *= 5 mice/group). (G) Expression of SHBs in pancreatic islets of LSL-SHBs and LSL-SHBs/Alb-Cre mice detected by immunofluorescence assay. The sections were immunostained for SHBs (red) and glucagon or insulin (green) as indicated. (H, I) The serum glucagon and insulin levels (H) and body weight (I) of LSL-SHBs and LSL-SHBs/Alb-Cre mice after 16 h fasting (*n *= 9 to 10 mice/group). (J) Hematoxylin-eosin staining on serial sections of livers (left) and the levels of serum ALT and AST (right, *n *= 10 mice/group) in LSL-SHBs and LSL-SHBs/Alb-Cre mice. Data are presented as means ± SEM. *, *P < *0.05; ns, no significant difference.

To further confirm the effect of SHBs on the stimulation of hepatic gluconeogenesis, we generated a liver-specific SHBs-expressing mouse line by crossing homozygous Rosa26^Loxp-Stop-Loxp-SHBs^ transgenic mice (LSL-SHBs) with albumin-Cre mice. The expression of SHBs was observed clearly in the liver of LSL-SHBs/Alb-Cre mice but not in the control heterozygous LSL-SHBs mice as detected by Western blot analysis and IHC (Fig. 3A). As expected, LSL-SHBs/Alb-Cre mice also exhibited a higher fasting blood glucose level and impaired tolerance to glucose and insulin (Fig. 3B to D). Again, the pyruvate tolerance was compromised, and the expression of *G6pc* and *PEPCK* was elevated in LSL-SHBs/Alb-Cre mice (Fig. 3E and F). We also checked the expression of SHBs in pancreatic islet alpha and beta cells by immunofluorescence staining. As anticipated, there was no SHBs in pancreatic islet cells (Fig. 3G). The differences in serum levels of glucagon or insulin were not discernible between the two groups (Fig. 3H), and the body weight, liver histology, and serum levels of ALT or AST were not significantly changed either (Fig. 3I and J). Taken together, these results suggest that the expression of SHBs in the liver may be a driving force for hepatic gluconeogenesis.

### SHBs induces hepatic gluconeogenesis via the PKA-CREB signaling pathway.

**FIG 4:**
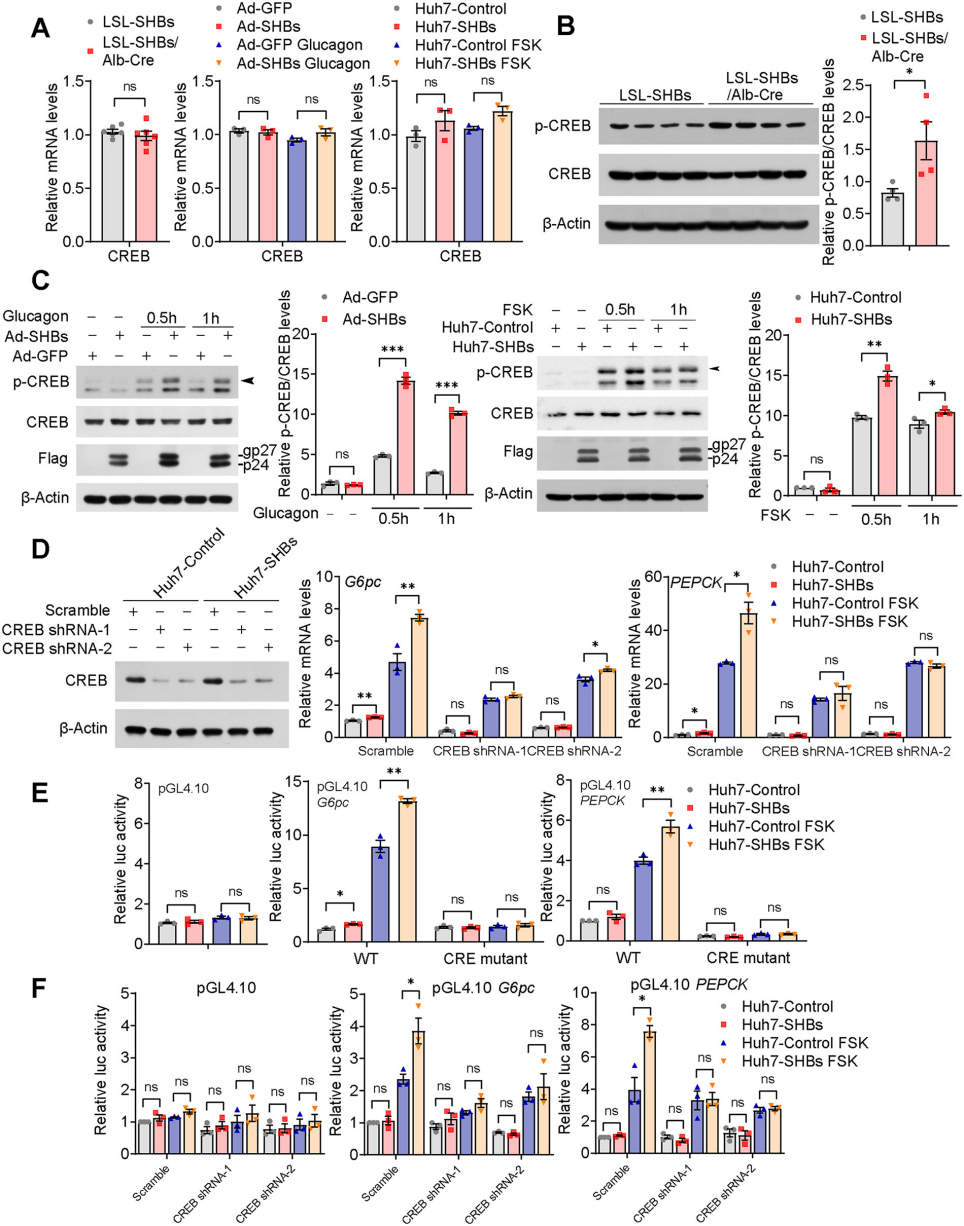
SHBs promotes hepatic gluconeogenesis via activation of CREB. (A) Relative mRNA expression levels of *CREB* in the livers of LSL-SHBs and LSL-SHBs/Alb-Cre mice after 16 h fasting (left, *n *= 5 mice/group), in the Ad-GFP and Ad-SHBs infected mouse primary hepatocytes after glucagon (100 nM) stimulation for 2 h (middle), and in Huh7-Control and Huh7-SHBs cells exposed to FSK (10 μM) for 2 h (right). Relative mRNA levels were determined by RT-qPCR. (B) Phosphorylated and total CREB levels in the livers of LSL-SHBs and LSL-SHBs/Alb-Cre mice after 16 h fasting, determined by Western blot analysis (*n* = 4 mice/group). (C) Western blot analysis of phosphorylated and total CREB levels of SHBs in SHBs-expressing mouse primary hepatocytes (left) and Huh7 cells (right) treated with glucagon (100 nM) or FSK (10 μM), respectively, for 0.5 h and 1 h. Arrowhead points to phospho-CREB. (D) Effect of CREB knockdown in Huh7-SHBs cells treated with 10 μM FSK for 2 h on *G6pc* and *PEPCK* mRNA expression. shRNA-mediated knockdown of CREB in Huh7-Control and Huh7-SHBs cells confirmed by Western blot analysis. (E, F) Effect of CRE mutation (E) and CREB knockdown (F) on promoter activities of *G6pc* and *PEPCK* in Huh7-SHBs cells. Huh7-Control and Huh7-SHBs cells were transfected with pRL-TK and the reporter genes. After 36 h transfection, cells were serum starved overnight and treated with FSK (10 μM) for 6 h. The dual-luciferase reporter gene assays were then carried out and the luciferase activity of the control group was normalized to 1. The glycosylated (gp) and nonglycosylated (p) forms of SHBs were indicated. Data are presented as means ± SEM. *, *P < *0.05; **, *P < *0.01; ***, *P < *0.001; ns, no significant difference.

We next sought to identify the signal component responsible for the SHBs-enhanced gluconeogenesis. CREB has been confirmed to the promoter of gluconeogenic genes, and its phosphorylation is an essential step in the regulation of gene expression of the enzymes of gluconeogenesis (24). Intriguingly we found that while SHBs did not affect CREB mRNA expression both *in vivo* and *in vitro* (Fig. 4A), CREB phosphorylation was markedly increased in the liver of the fasting LSL-SHBs/Alb-Cre mice compared with that of LSL-SHBs mice, with the total CREB protein levels unchanged (Fig. 4B). Furthermore, both the mouse primary hepatocytes infected with SHBs-expressing adenoviruses and Huh7-SHBs cells displayed significantly higher CREB phosphorylation than the control cells in response to glucagon or FSK (Fig. 4C). Consistently, knockdown of CREB abrogated SHBs-induced expression of gluconeogenic genes *G6pc* and *PEPCK* (Fig. 4D). It is known that CREB transactivates gluconeogenic gene expression by directly binding to the CREB response element (CRE) on the promoter of *G6pc* and *PEPCK* (25). As expected, SHBs increased the promoter activities of *G6pc* and *PEPCK*, which can be fully blocked when the binding sites of CRE to these two genes promoters were mutated (Fig. 4E). Moreover, knockdown of CREB also diminished the SHBs-enhanced promoter activities of *G6pc* and *PEPCK* (Fig. 4F). Therefore, the effect of SHBs on hepatic gluconeogenesis is likely to act through enhancing the phosphorylation of CREB.

**FIG 5:**
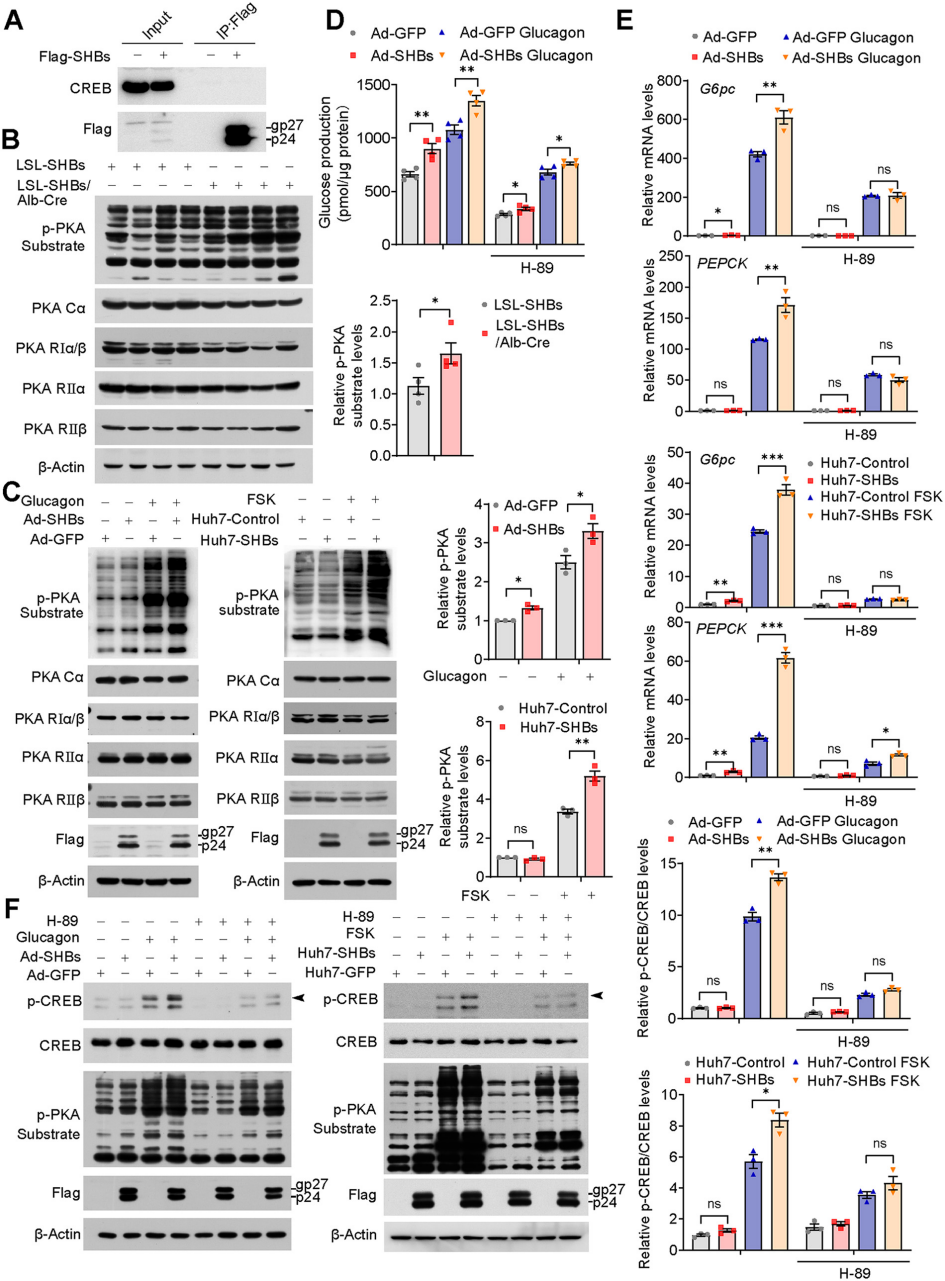
SHBs promotes activities of PKA. (A) Huh7 cells were transfected with pcDNA 3.1-SHBs-Flag for 48 h, and then a coimmunoprecipitation (co-IP) assay was performed to determine the interaction between SHBs and CREB. The immunoprecipitated complexes with anti-Flag antibody were subjected to immunoblotting with CREB antibody. (B, C) PKA activity and protein levels in the livers of LSL-SHBs and LSL-SHBs/Alb-Cre mice fasted for 16 h (B, *n* = 4 mice/group), in Ad-GFP and Ad-SHBs-infected mouse primary hepatocytes in the presence or absence of glucagon (100 nM) stimulation for 30 min, and in Huh7-Control and Huh7-SHBs cells exposed to FSK (10 μM) for 30 min (C). (D) Effect of PKA inhibitor H-89 on glucose production in Ad-SHBs-infected mouse primary hepatocytes. After a 36-h infection, cells were serum starved overnight and treated with H-89 (10 μM) for 1 h and then with glucagon (100 nM) for 6 h. (E, F) Effect of PKA inhibitor H-89 on gluconeogenic gene expression (E) and CREB phosphorylation and PKA activity (F) in Ad-SHBs-infected mouse primary hepatocytes and Huh7-SHBs cells. After being serum starved overnight, cells were treated with H-89 (10 μM) for 1 h before 2 h (E) and 30 min (F) glucagon (100 nM) or FSK (10 μM) stimulation, respectively. The glycosylated (gp) and nonglycosylated (p) forms of SHBs were indicated. Arrowhead points to phospho-CREB. Data were expressed as means ± SEM. *, *P < *0.05; **, *P < *0.01; ***, *P < *0.001; ns, no significant difference.

To investigate whether there was a direct interaction between SHBs and CREB, coimmunoprecipitation was performed and showed no physical interaction between SHBs and CREB (Fig. 5A). PKA functions upstream of CREB and is a holoenzyme consisting of four subunits, as follows: two catalytic and two regulatory subunits (26). Although the expression levels of PKA subunits (PKA-Cα, -RIα/β, -RIIα, and -RIIβ) were about the same, PKA activity in the liver of the fasting LSL-SHBs/Alb-Cre mice was markedly increased, as evidenced by a higher phosphorylation of PKA substrate compared with the control (Fig. 5B). Consistent with the results obtained *in vivo*, SHBs also significantly increased PKA activity in both glucagon-stimulated mouse primary hepatocytes and FSK-stimulated Huh7 cells without a change in PKA total protein levels (Fig. 5C). To further confirm that PKA was involved in the SHBs-induced upregulation of gluconeogenesis, PKA inhibitor H-89 was employed and the results showed that H-89 attenuated SHBs-stimulated glucose production in the mouse primary hepatocytes (Fig. 5D). SHBs-enhanced gluconeogenic genes expression (Fig. 5E), CREB phosphorylation, and PKA activity (Fig. 5F) were also abrogated in H-89-treated mouse primary hepatocytes and Huh7 cells. Taken together, these data support the notion that SHBs promote hepatic gluconeogenesis through PKA-mediated CREB phosphorylation.

### SHBs increases in cAMP production in hepatocytes depends on adenylate cyclase.

**FIG 6:**
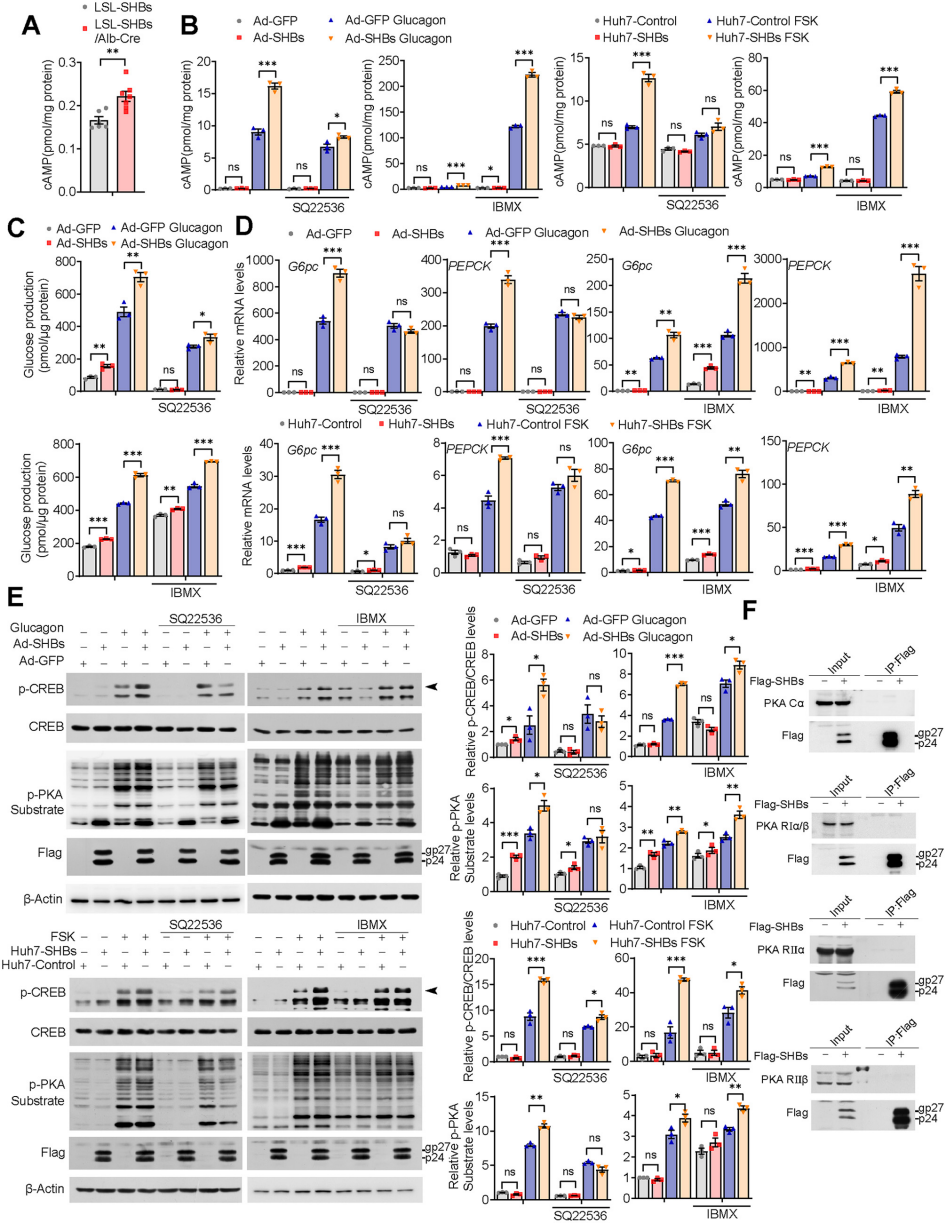
SHBs elevates cAMP levels by regulating AC. (A) cAMP levels in the liver of LSL-SHBs and LSL-SHBs/Alb-Cre mice fasted for 16 h (*n* = 6 to 7 mice/group). (B to E) Effect of AC inhibitor SQ22536 or PDE inhibitor IBMX on cAMP levels (B), glucose production (C), gluconeogenic gene expression (D), CREB phosphorylation, and PKA activity (E) in Ad-SHBs-infected mouse primary hepatocytes and in Huh7-SHBs cells. Cells were treated with SQ22536 (500 μM) or IBMX (500 μM) for 1 h and then with glucagon (100 nM) stimulation or FSK (10 μM) for 15 min (B), 6 h (C), 2 h (D) and 30 min (E). (F) Huh7 cells were transfected with pcDNA 3.1-SHBs-Flag for 48 h, and then co-IP assays were performed to determine the interaction between SHBs and PKA subunits. The immunoprecipitated complexes with anti-Flag antibody were subjected to immunoblotting with PKA Cα, PKA RIα/β, PKA RIIα, or PKA RIIβ antibody. The glycosylated (gp) and nonglycosylated (p) forms of SHBs were indicated. Arrowhead points to phospho-CREB. Data are presented as means ± SEM. *, *P < *0.05; **, *P < *0.01; ***, *P < *0.001; ns, no significant difference.

The expression of glucose-producing enzymes is tightly controlled by the glucagon/cAMP/PKA pathway (27). An increase of intracellular cAMP, a second messenger transferring the signaling of glucagon, results in activation of PKA (26). We found that cAMP levels in the liver of the fasting LSL-SHBs/Alb-Cre mice was increased compared with those in the controls (Fig. 6A). Also, SHBs expression in the mouse primary hepatocytes significantly elevated intracellular cAMP levels in response to glucagon stimulation, and the same was true for the Huh7-SHBs cells under FSK treatment (Fig. 6B). Since the intracellular cAMP level is fine-tuned by adenylyl cyclase (AC) for its production and by phosphodiesterase (PDE) for its degradation (26), we assumed that SHBs-enhanced cAMP levels may be mediated by activating AC or inhibiting PDE or both. A selective AC inhibitor, SQ22536, and a pan PDE inhibitor, 3-isobutyl-1-methylxanthine (IBMX), were applied to explore whether AC and/or PDE was responsible for the increased cAMP by SHBs. Exposure to SQ22536 caused a significant attenuation of intracellular cAMP initially increased by SHBs (Fig. 6B). Moreover, SQ22536 also suppressed glucagon-stimulated glucose production, *G6pc* and *PEPCK* mRNA expression, CREB phosphorylation and PKA activity in Ad-SHBs-infected mouse primary hepatocytes and in Huh7-SHBs cells under FSK treatment (Fig. 6C to E). With respect to IBMX, while IBMX *per se* could increase cAMP levels by its default action and subsequently enhanced glucagon-stimulated glucose production, gluconeogenic gene expression, CREB phosphorylation, and PKA activity, SHBs still retained its effects on these parameters (Fig. 6B to E). Clearly, it is AC playing an important role in mediating SHBs’s regulation of intracellular cAMP concentration and subsequently the promotion of PKA/CREB signaling and gluconeogenic gene expression. Of note, there was no interaction between SHBs and any of PKA subunits, as shown by the coimmunoprecipitation study (Fig. 6F).

### SHBs enhances hepatic expression and promoter activity of AC1.

**FIG 7:**
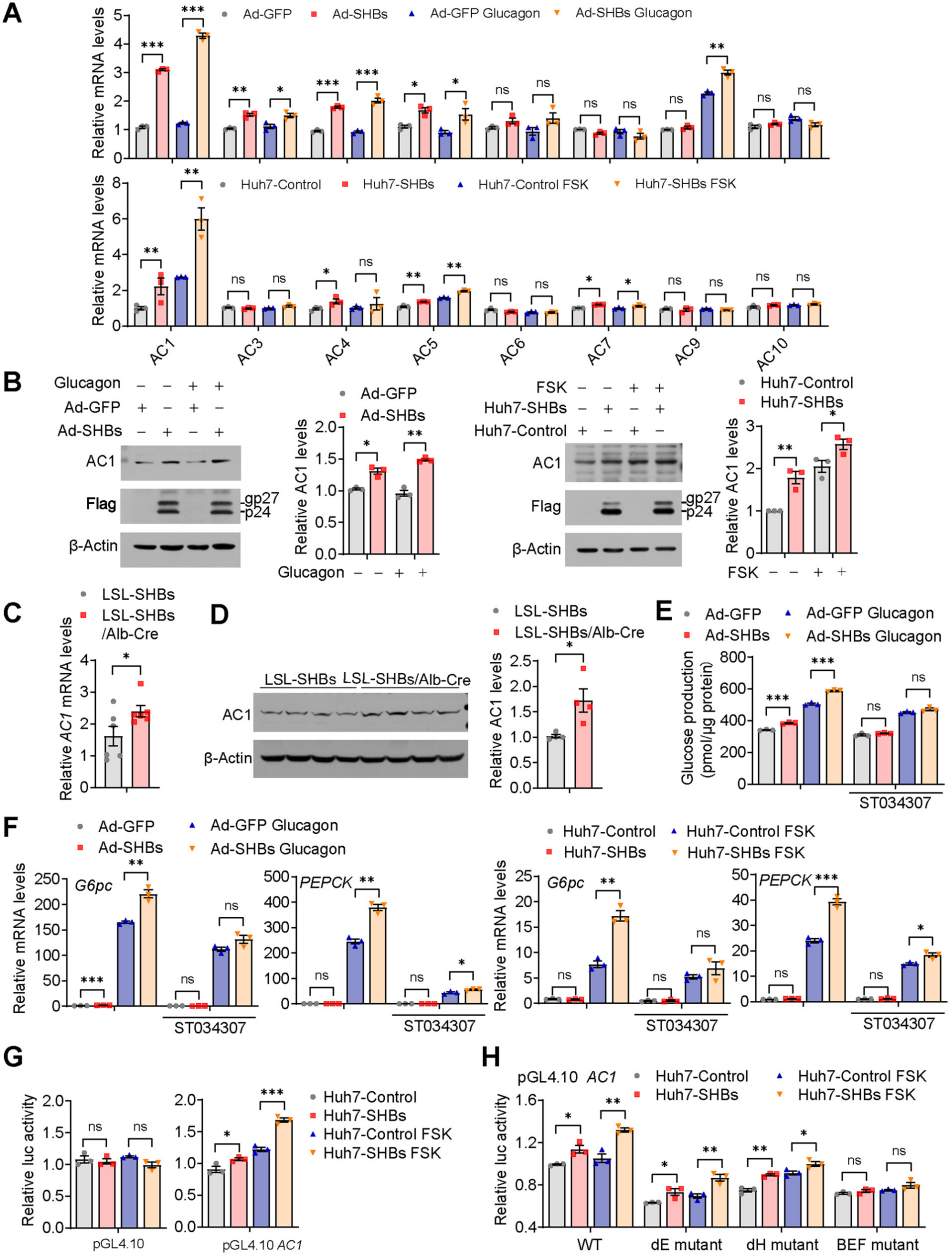
SHBs elevates hepatic AC1 expression. (A) Relative mRNA expression levels of ACs in Ad-GFP and Ad-SHBs-infected mouse primary hepatocytes exposed to glucagon (100 nM, top) and in Huh-Control and Huh7-SHBs cells exposed to FSK (10 μM, bottom) for 2 h determined by RT-qPCR. (B) Effect of SHBs on AC1 protein levels in mouse primary hepatocytes exposed to glucagon (100 nM, left) and Huh7 cells exposed to FSK (10 μM, right) for 16 h detected by Western blot analysis. (C, D) The mRNA and protein levels of AC1 in the livers of LSL-SHBs and LSL-SHBs/Alb-Cre mice fasted for 16 h determined by RT-qPCR (C, *n *= 6 mice/group) and Western blot analysis (D, *n* = 4 mice/group). (E, F) Effect of AC1 specific inhibitor ST034307 on glucose production (E) and gluconeogenic gene expression (F) in Ad-SHBs-infected mouse primary hepatocytes and in Huh7-SHBs cells. Cells were treated with ST034307 (20 μM) for 1 h and then with glucagon (100 nM) or FSK (10 μM) for 6 h (E) and 2 h (F). (G) Effect of SHBs on promoter activities of *AC1* in Huh7 cells. Huh7-Control and Huh7-SHBs cells were transfected with pRL-TK and the reporter genes. After 36 h of transfection, cells were serum -starved overnight and treated with FSK (10 μM) for 6 h. The dual-luciferase reporter gene assays were then carried out, and the luciferase activity of the control group was normalized to 1. (H) Effect of E-box mutant (dE), nuclear receptor binding half site mutant (dH), and BEF mutant on promoter activity of AC1 in Huh7-SHBs cells. The glycosylated (gp) and nonglycosylated (p) forms of SHBs were indicated. Data are presented as means ± SEM. *, *P < *0.05; **, *P < *0.01; ***, *P < *0.001; ns, no significant difference.

There are nine transmembrane and one soluble AC isoforms in mammals, each with distinct expression patterns in the digestive system (28). Except for AC2 and AC8, all isoforms of AC are expressed in hepatocytes (29). Interestingly, in both mouse primary hepatocytes and Huh7 cells, gene expression analysis revealed that SHBs had the largest effect on increasing AC1 mRNA levels among all isoforms tested (Fig. 7A). Furthermore, the protein levels of AC1 were increased in Ad-SHBs-infected mouse primary hepatocytes and Huh7-SHBs cells (Fig. 7B). Consistent with the results obtained *in vitro*, SHBs elevated the mRNA and protein levels of AC1 in the liver of LSL-SHBs/Alb-Cre mice (Fig. 7C and D). The role of AC1 in SHBs-mediated regulation of hepatic gluconeogenesis was further verified using a specific AC1 inhibitor, ST034307. The results showed that ST034307 attenuated glucagon-induced glucose production and mRNA expression of *G6pc* and *PEPCK* in Ad-SHBs-infected mouse primary hepatocytes and Huh7-SHBs cells under FSK treatment (Fig. 7E and F). We examined the ability of SHBs to activate the transcription activity of AC1, and the mouse AC1 promoter (−476/+65) was constructed into a pGL4.10 reporter plasmid and transfected in Huh7-SHBs cells. As shown in Fig. 7G, AC1 promoter activity was enhanced by SHBs. A 15-bp binary E-box factor binding site (BEF), which was composed of a binding half site for nuclear hormone receptor superfamily (H) and a binding site for E-box binding factors (E), in the promoter of AC1 was reported to play a major role in AC1 expression regulation in neurons (30). We assessed the possibility that SHBs activated AC1 transcription through BEF. Mutations of E or H alone obviously decreased the promoter activity of AC1; however, they could not completely block the effect of SHBs (Fig. 7H). In contrast, when all the 15 bp of BEF was mutated, SHBs lost its ability to elevate AC1 promoter activity (Fig. 7H). Collectively, these data indicate that the activation of cAMP/PKA/CREB signaling and enhanced hepatic gluconeogenesis by SHBs are largely dependent on its ability to induce transcription activity and expression of AC1 through the BEF.

## DISCUSSION

**FIG 8:**
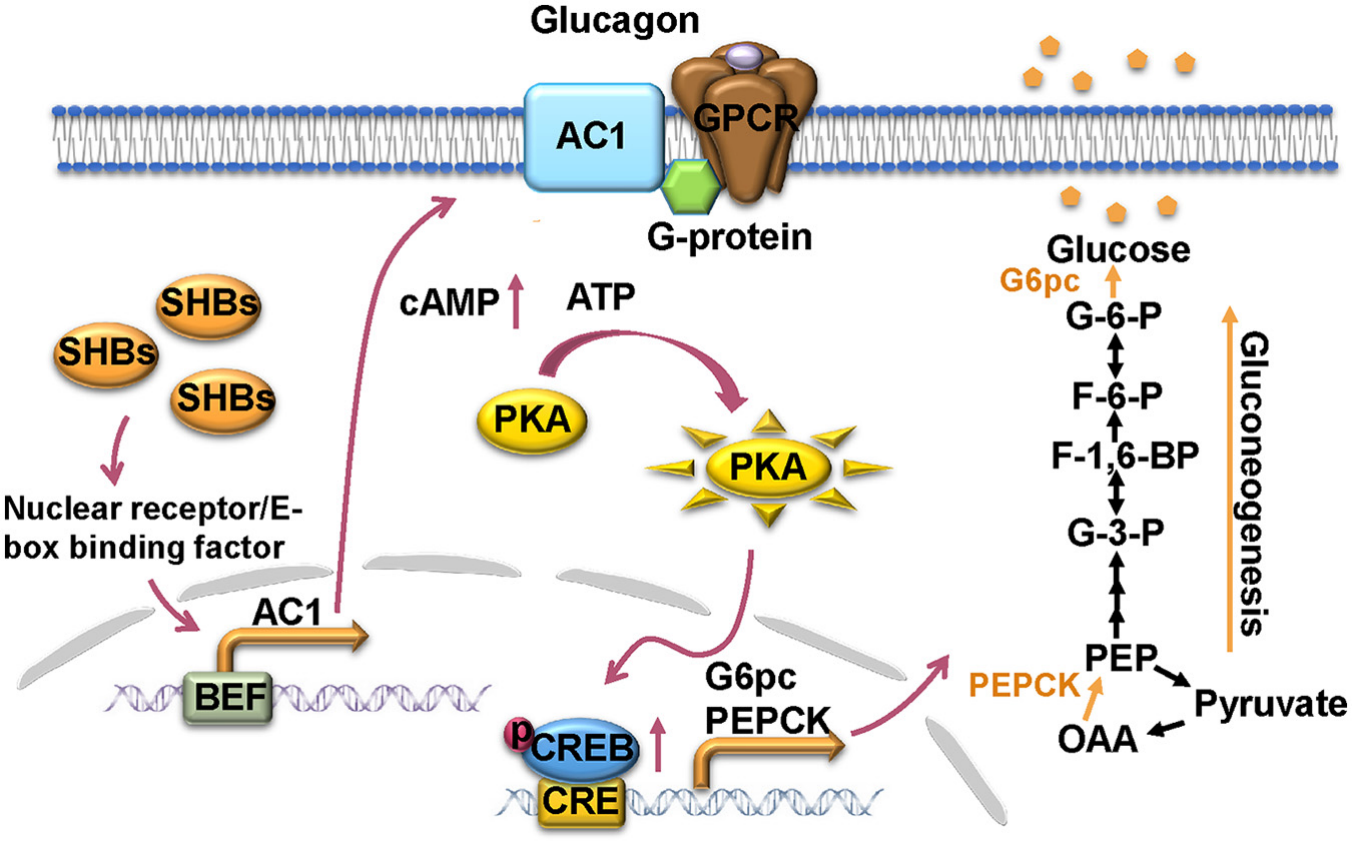
A working model demonstrating the regulation of SHBs on hepatic gluconeogenesis. In the liver, SHBs enhances glucagon-induced cAMP/PKA/CREB signaling through elevation of nuclear receptor/E-box binding factor/BEF-mediated AC1 transcription, subsequently activating CREB downstream gluconeogenic genes *G6pc* and *PEPCK* and consequently promoting hepatic gluconeogenesis.

HBV is a hepatotropic virus whose infection leads to a series of liver diseases, such as hepatitis, cirrhosis, and even hepatocellular carcinoma (15). The liver is an important organ of the body that maintains the blood glucose levels, owing to its ability to regulate glucose homeostasis. Diabetes is closely linked to liver diseases. Patients with type 2 diabetes have a high prevalence of nonalcoholic fatty liver disease (NAFLD), nonalcoholic steatohepatitis (NASH), and advanced fibrosis (31). Except NAFLD, diabetes also represents an important risk factor for hepatocellular carcinoma (32, 33). Hepatitis C infection is reported to induce insulin resistance and be associated with an increased risk of type 2 diabetes (34, 35). Many studies have provided evidence for the association between HBV and diabetes (2–6, 36). Although some studies suggest that the increased risk of diabetes in HBV infection patients was associated with HBV-related cirrhosis rather than itself (37), in a large population study, the association of HBV infection with diabetes was still prominent even after excluding patients with cirrhosis (38). High viral load and long duration of chronic hepatitis B have been identified as potential risk factors of type 2 diabetes in patients with HBV infection (39). Moreover, HBx has been reported to disturb hepatic glucose homeostasis through inducible nitric oxide synthase (iNOS)-mediated Jun N-terminal protein kinase (JNK) activation (20). Therefore, cirrhosis may not be the only prerequisite for the HBV-associated high prevalence of diabetes, and there should exist the possibility that HBV itself or its viral proteins could directly cause abnormal glucose metabolism and a subsequent development of diabetes. In this study, we provide compelling evidence that the small surface antigen of HBV (SHBs), the most abundant viral protein of HBV, promotes hepatic gluconeogenesis by upregulation of AC1 transcription and expression to activate cAMP/PKA/CREB signaling for the induction of gluconeogenesis enzyme gene expression (Fig. 8).

We first demonstrated that SHBs is a positive regulator of hepatic gluconeogenesis. Ectopic expression of SHBs increased glucose production and gluconeogenic gene expression in hepatocytes, which was independent of its glycosylation status. Both AAV8-delivered hepatic SHBs overexpression mice and liver-specific SHBs transgenic mice exhibited elevated fasting blood glucose levels and impaired glucose and insulin tolerance. Abnormal hepatic gluconeogenesis is a major contributor to hyperglycemia and glucose intolerance in diabetes (9). We found that the pyruvate tolerance was compromised and gluconeogenic genes were upregulated by SHBs in the mice. It is noteworthy that secreted SHBs did not alter glucose production and gluconeogenic gene expression in the hepatocytes. Taken together, these data suggest that intracellular SHBs affects glucose homeostasis likely through promoting hepatic gluconeogenesis.

Hepatic gluconeogenesis is positively regulated by glucagon. The hyperglycemic actin of aberrantly secreted glucagon is recognized as an important mechanism for the pathogenesis of diabetes (40). Activation of key transcription factor CREB by phosphorylation at Ser133 is a critical step in glucagon-stimulated hepatic gluconeogenesis (27). It has been shown that hepatitis B virus X protein (HBx) serves as a transcriptional coactivator and interacts directly with CREB (41, 42). Moreover, HBV DNA polymerase binds to and stabilizes CREB mRNA and increases its expression (43). In the current study, we demonstrated that SHBs markedly increased the phosphorylation of CREB at Ser133 *in vitro* and *in vivo* without affecting mRNA and protein expression of CREB. Phosphorylation of CREB at Ser133 is mediated by PKA in response to glucagon (27). Consistent with CREB phosphorylation, PKA activity was also elevated by SHBs *in vitro* and *in vivo*. Pharmacological inhibition of PKA attenuated the effect of SHBs on glucose production, gluconeogenic gene expression, and CREB phosphorylation. Taken together, these results pinpoint a pivotal role of PKA/CREB signaling in SHBs-induced hepatic gluconeogenesis.

In the rest state, PKA is an inactivated heterotetramer with two catalytic and two regulatory subunits in the cytoplasm. Once glucagon binding with GCGR, cAMP is accumulated and binds to the PKA regulatory subunits, thus liberating catalytic subunits of PKA for entry into the nucleus to phosphorylate nuclear CREB (26). cAMP is the initial signal for PKA activation in response to glucagon. We found that SHBs significantly increased cAMP levels under glucagon or FSK treatment. When the inhibitors of AC and PDE were applied, it was found that only an AC but not an PDE inhibitor could affect SHBs-associated regulation of hepatic gluconeogenesis and cAMP/PKA/CREB signaling. After screening of all the isoforms of AC in hepatocytes, we found that SHBs had the largest effect on the upregulation of the AC1 isoform. Inhibition of AC1 attenuated the effect of SHBs on glucagon-induced hepatic gluconeogenesis. AC1 has been reported to express highly in the brain and regulate neuronal signal transduction and synaptic plasticity (44). Except for the brain, gene expression of AC1 is higher in the liver than in other tissues based on data from the Genotype-Tissue Expression (GTEx) project (45). We further found SHBs upregulated AC1 transcription activity to elevate its expression. The transcription activity of AC1 is enhanced by the BEF in its promoter in neurons (30). BEF is a composite binding site for nuclear hormone receptors and E-box binding factors. The BMAL1/CLOCK complex and NPAS2/BMAL1 complex were found to activate AC1 expression by binding to E-box within BEF (46, 47). In the current study, only the BEF mutant but not the E-box mutant or nuclear receptor binding half site mutant impaired the enhancement effect of SHBs on AC1 promoter activity, suggesting that both the nuclear receptor and E-box factor binding to BEF are crucial to the elevation capacity of SHBs. Taken together, our study indicates that the effect of SHBs on hepatic gluconeogenesis is mediated mainly through upregulation of AC1 transcription activity through BEF and uncovers a potential function of AC1 outside the brain.

SHBs is synthesized from HBV subgenomic preS2/S mRNA in the endoplasmic reticulum (ER) (48). We and others have shown that SHBs accumulation induces ER stress, which contributes to the progression of several liver diseases (17, 23, 49, 50). For instance, SHBs-induced ER stress promotes metastasis and angiogenesis of hepatocellular carcinoma (HCC) (23, 49). To investigate whether ER stress is involved in the upregulation of AC1 expression by SHBs, the ER stress inhibitor tauroursodeoxycholic acid (TUDCA) was used. However, under the treatment of TUDCA, Ad-SHBs-infected mouse primary hepatocytes and Huh7-SHBs cells still exhibit a higher AC1 mRNA level and promoter activity than respective controls (data not shown). Therefore, it suggested that ER stress may not be involved in SHBs regulation of hepatic gluconeogenesis and the mechanism of how SHBs modulates AC1 transcription activity through nuclear receptor/E-box binding factor and BEF needs to be further studied.

In summary, this study has revealed a novel role of SHBs in the regulation of hepatic gluconeogenesis via activation of cAMP/PKA/CREB signaling, which may provide new mechanistic insight into the possible link between HBV infection and diabetes and may also offer potential preventive and therapeutic targets for glucose metabolism disorder in HBV patients.

## MATERIALS AND METHODS

### Animal experiments.

All animal experiments were approved by the Institutional Animal Care and Use Committee of Fujian Medical University. All animals were maintained in ventilated cages under a 12-h light/dark cycle with free access to food and water. Male wild-type C57BL/6 mice were purchased from GemPharmatech Co., Ltd. (Nanjing, China). They were administered with a purified liver-targeted adeno-associated virus 8 (AAV8) carrying SHBs-Flag (2 × 10^11^ vector genomes) by tail vein injection to achieve an overexpression of SHBs in the liver. The control group was injected with AAV8-Control. AAV8 was used because of its higher affinity for liver. These adeno-associated viruses were purchased from Obio Technology (Shanghai, China).

Rosa26^Loxp-Stop-Loxp-SHBs^ transgenic C57BL/6 mice (LSL-SHBs) were generated by Shanghai Biomodel Organism Science & Technology Development Co., Ltd. (Shanghai, China). Homozygous LSL-SHBs mice were bred with albumin-Cre mice to generate liver-specific SHBs-overexpressed (LSL-SHBs/Alb-Cre) mice where SHBs was transcriptionally activated after Cre-mediated excision of a loxP-flanked Stop cassette. Heterozygous LSL-SHBs mice served as the control.

Blood glucose levels were measured using a OneTouch UltraVue glucometer (Johnson & Johnson, New Brunswick, NJ). The levels of insulin and glucagon in serum were measured using enzyme-linked immunosorbent assay (ELISA) kits (USCN Life Science Inc, Wuhan, China). Mice were fasted for 16 h and then subjected to glucose tolerance tests (GTTs) or pyruvate tolerance tests (PTTs). Insulin tolerance tests (ITTs) were performed in mice fasted for 5 h. Mice were injected intraperitoneally with 2 g/kg of body weight of glucose for GTT, 2 g/kg of sodium pyruvate for PTT, or 0.5 U/kg of insulin for ITT, and then tail vein blood was collected at specific time intervals for the measurement of blood glucose. Serum levels of alanine transaminase (ALT) and aspartate transaminase (AST) were determined using a standard clinical automatic analyzer (7020; Hitachi, Kyoto, Japan).

### Plasmid constructions.

pcDNA3.1-SHBs-Flag was used as described previously (17). pcDNA3.1-SHBs-Flag was constructed by inserting a PCR-generated SHBs-Flag sequence into the HindIII/Not I recognition sites of pcDNA3.1/Hygro(+) (Invitrogen, Carlsbad, CA). The HBV DNA (3,215 bp, genotype B, adw subtype, GenBank accession number AF100309) was used as a template. pRep-HBV harboring 1.2-U lengths of the HBV genome and control plasmid pRep-Sal I were described previously (51). The pRep-HBV-SHBs (−) mutant contains ACG instead of the SHBs start codon. The pRep-HBV-HBx (−) mutant contains a stop codon at HBx codon 8 (CAA to TAA). These two mutants were generated as described previously (52). The human *G6pc* promoter (−1247/+83) and *PEPCK* promoter (−1968/+112) were amplified by PCR and inserted into the pGL4.10 vector using KpnI/XhoI and XhoI/BglII recognition sites, respectively. The CREB response element (CRE) mutants of pGL4.10-*G6pc* and pGL4.10-*PEPCK* were generated by ligation-independent cloning (LIC). Primers are provided in Table 1. The mouse *AC1* promoter (−476/+65) and its mutants, including E-box mutant (dE), nuclear receptor binding half site mutant (dH), and BEF mutant, were synthesized chemically and provided by General Biosystems (Anhui, China) with KpnI/XhoI recognition sites on two ends and then cloned into the pGL4.10 vector. E-box was mutated from 5′-CACGTG-3′ to 5′-TGCGCA-3′. The nuclear receptor binding half site was mutated from 5′-AGGTCA-3′ to 5′-GTAACA-3′. BEF was mutated from 5′-CCAAGGTCACGTGGC-3′ to 5′-ATCGTAGTCTTCTAA-3′.

**TABLE 1:** Primers used for pGL4.10 reporter plasmids constructions

Gene	Primer sequence (5′–3′) by direction^*a*^	
	Forward	Reverse
*G6pc* promoter	CTGGTACCAGCCAAGACTGGCAGATCTCT	CGCTCGAGCATCCTCATTTCCTTGGCACCT
*PEPCK* promoter	GCCTCGAGTTGACAAGGGACAGTTGC	CGCAGATCTTGGATGATCTCGAAGGGA
*G6pc* CRE mutant		
Sequence 1	CCTCTAGACTCGAGGCCACCATGGAGGAAGGAATGAATGTTC	GTAGGTTGTCCCAAACATGTTCAGGGTGATTTAGCAAAAATAGAAAAACAGGC
Sequence 2	CTATTTTTGCTAAATCACCCTGAACATGTTTGGGACAACCTACTGGTGATGCA	CCGAGCTCGGATCCCAACGACTTCTTGTGCGGCTGG
*PEPCK* CRE mutant		
Sequence 1	TCGCTAGCCTCGAGTTGACAAGGGACAGTTGC	CGCCACTGTTTTCAGGGGGCAGGTCTCTG
Sequence 2	CCCCCTGAAAACAGTGGCGAGCCTCCCTG	CCGAGGCCAGATCTTGGATGATCTCGAAGGGA

a Mutated sites are marked with an underline.

### Cell culture and treatment.

The human embryonic kidney 293T and HEK293 cell line was obtained from the American Type Culture Collection (ATCC, MD) and cultured in Dulbecco’s modified Eagle’s medium (DMEM) with 10% fetal bovine serum (FBS; PAN-Biotech GmbH, Aidenbach, Germany). HepAD38 cells were purchased from the Shanghai Second Military Medical University and maintained in DMEM with 10% FBS, 3 μg/mL doxycycline, and 400 μg/mL G418 (Sigma-Aldrich, St. Louis, MO). HBV virions were collected from the supernatant of HepAD38 cells after a withdrawl of doxycycline. Primary human hepatocytes (PHHs) were purchased from Liver Biotechnology Co., Ltd. (Shenzhen, China). The cells were cultured in the maintenance medium (Liver Biotechnology Co., Ltd.) supplemented with Forskolin (20 μM), SB431542 (10 μM), IWP2 (0.5 μM), DAPT (5 μM), and LDN193189 (0.1 μM) for long-term functional maintenance *in vitro* (53), and they were infected with HBV virions at a multiplicity of infection (MOI) of 1,000 viral genome equivalents (VGE) per cell in the presence of 2% dimethyl sulfoxide (DMSO) and 4% polyethylene glycol 8000 (PEG 8000). After 16 h, cells were washed with PBS three times and then cultured in the maintenance medium. Mouse primary hepatocytes were isolated from 4-week-old C56BL/6 male mice using type IV collagenase (Sigma-Aldrich, St. Louis, MO) and cultured in DMEM with 10% FBS. For SHBs overexpression, mouse primary hepatocytes were infected with the adenoviruses expressing SHBs (Ad-SHBs) for 36 h. Then, the mouse primary hepatocytes were serum starved overnight before 100 nM glucagon treatment. Huh7 was purchased from China Center for Type Culture Collection (Shanghai, China) and cultured in DMEM with 10% FBS. SHBs stably expressing Huh7 cells (Huh7-SHBs) and its control (Huh7-Control) were generated by transfection with pcDNA3.1-SHBs-Flag and empty plasmid, respectively. Cells were selected under 400 μg/mL hygromycin for 4 weeks and subjected to Western blot analysis to confirm SHBs expression. Huh7-SHBs and Huh7-control cells were serum starved overnight before forskolin (FSK) treatment. Transfection was performed using Lipofectamine 3000 transfection reagent (Invitrogen) according to the manufacturer’s instructions. A variety of inhibitors were applied 1 h prior to glucagon or FSK.

### Recombinant adenovirus and lentivirus.

Ad-SHBs was produced and supplied by WZ Bioscience Inc. (Shandong, China). Ad-SHBs N146Q was generated by the AdEasy adenoviral vector system (Stratagene, La Jolla, CA). HEK293 cells were transfected with linearized recombinant adenovirus DNA. After 10 days, HEK293 cells were collected and lysed by three freeze-thaw-vortex cycles. The recombinant adenoviruses were stepwise amplified by reinfecting HEK293 cells with cell lysates. The SHBs and SHBs-N146Q were fused with Flag tag sequences. CREB knockdown was performed by using CREB-specific short hairpin RNA (shRNA) lentiviral particles. A shRNA sequence that did not target any known human gene served as a scrambled negative control. shRNA sequences (Invitrogen, Carlsbad, CA) were annealed and subcloned into lentiviral vector pLL3.7. Lentiviruses were produced by cotransfecting subconfluent HEK293T cells with the lentiviral vector and packaging plasmids. Viral supernatants were collected 48 h after the transfection and filtered through 0.45-μm filters (EMD Millipore, Billerica, MA). Freshly plated cells were then infected with the lentiviruses for 48 h and selected by puromycin. The knockdown efficiency was determined by Western blot analysis. The shRNA sequences were as follows: 5′-GCAGCTCATGCAACATCAT-3′ (CREB-shRNA1), 5′-GCCAACTCCAATTTACCAA-3′ (CREB-shRNA2), and 5′-GCGCGCTTTGTAG GATTCG-3′ (scramble-shRNA).

### RNA interference.

For knockdown of SHBs in Huh7-SHBs cells, the small interfering RNA (siRNA) specifically targeting SHBs was designed and synthesized chemically by Shanghai GenePharma Co. (Shanghai, China). A nontargeting siRNA (NC-siRNA) was used as a negative control. Huh7-SHBs cells were transfected with siRNA for 48 h, and then the gene silencing efficiency was confirmed by Western blot analysis. The siRNA sequences were as follows: 5′-CAUCACAUCAGGAUUCCUATT-3′ (SHBs-siRNA1), 5′-CCUCCAAUCACUCACCAA CTT-3′ (SHBs-siRNA2), and 5′-UUCUCCGAACGUGUCACGUTT-3′ (NC-siRNA).

### Glucose production assay.

For the glucose production assay, primary hepatocytes were incubated in glucose and phenol-red-free DMEM supplemented with gluconeogenic substrates (20 mM sodium lactate and 2 mM sodium pyruvate) in the presence or absence of glucagon (100 nM) for 6 h. Then the medium was collected to determine glucose production using the high-sensitivity glucose assay kit (Sigma-Aldrich), and glucose levels were normalized to total protein levels.

### cAMP measurement.

After overnight serum starvation, Ad-SHBs-infected mouse primary hepatocytes or Huh7-SHBs cells were treated with 100 nM glucagon or 10 μM FSK, respectively, for 15 min and then assayed using a cAMP ELISA kit (Cell Biolabs, Inc., San Diego, CA). The cAMP levels in the liver of 16-h fasting mice were also measured. The cAMP levels were normalized to total protein levels.

### Real-time quantitative PCR (RT-qPCR).

Total RNA was extracted using the TRIzol reagent (Invitrogen) and was used for cDNA synthesis by HiScript III RT SuperMix for qPCR with genomic DNA (gDNA) wiper (Vazyme, Nanjing, China). Real-time PCR was performed on a Mx3000P real-time PCR system (Agilent Technologies, Santa Clara, CA) with the ChamQ Universal SYBR qPCR master mix (Vazyme) following the manufacturer’s instructions. The primers used are listed in Table 2.

**TABLE 2:** Primers used for quantitative real-time PCR

Gene by organism	Primer sequence (5′–3′) by direction	
	Forward	Reverse
For mouse		
*G6pc*	CTGTCCCGGATCTACCTTGC	TTGTAGATGCCCCGGATGTG
*PEPCK*	ATGAAAGGCCGCACCATGTA	GCACAGATATGCCCATCCGA
*CREB*	ACCCAGGGAGGAGCAATACAG	TGGGGAGGACGCCATAACA
*AC1*	TTGGCAAGTTCGATGAGTTAGC	GGCGTGATCCGTCTTAGGC
*AC3*	ATGTCACCGTGGCAAACAAGA	GCAATGATGAGGTAGGTTTCGAT
*AC4*	GTCCTTGGACTGTATCTTGGGT	CCACACAGGAACAATACCGC
*AC5*	AACGCCAAGCAGGAGGATATG	CCCCGAGGATCTTAATCCGTAA
*AC6*	GATGAACGGAAAACAGCTTGGG	GGTGGCTCCGCATTCTTGA
*AC7*	CTCATGGTACTAGGCTCCGTG	GCTGAGAGGCAGTAGTGCATA
*AC9*	TAAGACCAGCACCAAGGCTTC	GTTCTTGAACCTGAGCGGGA
*AC10*	GTGGAAAGTGGAACGAAAGCA	GCCCTATCTTAACTCGAATGTCC
*β-actin*	GATGGCCACTGCCGCATCCTC	GGTCTTTACGGATGTCAACGTCAC
For human		
*G6pc*	TGAGTTGACCGAGAGTCCCA	AGTGACCCTGCCCATCTACT
*PEPCK*	TCCGGAAGGTGTTCCCATTG	GCCTTTATGTTCTGCAGCCG
*CREB*	GGCTCCAGATTCCATGGTC	GTGTTACGTGGGGGAGAGAA
*AC1*	CAGTACGACGTGTGGTCCAA	ACGCTAGGGTCGTCTTTGTG
*AC3*	GGAATTGGACTGGTGTTGGAC	GATCTGGGCGGTTATGAGCA
*AC4*	ACCTGGCCCGAGAGATGAA	CAGCTCCTTAGGGGAACACTC
*AC5*	GATCGAGGCCATCTCGTTGG	CGTGACATCGTTAGACCAGAC
*AC6*	CTCCTGGTCCCTAAAGTGGAT	GGAGGCAGCTCATATAGCGG
*AC7*	GGTGCTCGGTTCTTTGATGG	ATGCACTTCACAGTGTAGGTG
*AC9*	AAAAACAGCACCAAGGCTTCT	GAACCTCAGCGGAAGGAGAG
*AC10*	ACAAAGTGTACGACCTTCATGC	CGAAGCTCAGATAAATAGCCCTG
*β-actin*	GTCATTCCAAATATGAGATGCGT	GCTATCACCTCCCCTGTGTG

### Coimmunoprecipitation and Western blot analysis.

Coimmunoprecipitation and Western blot analysis were performed as described previously (49). The primary antibodies used in this studies included anti-Flag (F1804; Sigma-Aldrich), anti-SHBs (DMABT-51328MH; Creative-Diagnostics, Shirley, NY), anti-CREB (9197S; Cell Signaling Technology [CST], Beverly, MA), anti-phospho-CREB (9198S; CST), anti-phospho-PKA substrate (9624S; CST), anti-PKA Cα (4782S; CST), anti-PKA RIα/β (3927; CST), anti-β-Actin (3700; CST), anti-PKA RIIα (MAB8000; R&D Systems, Minneapolis, MN), anti-PKA RIIβ (PAB18374; Abnova Inc., Walnut, CA), and anti-AC1 (LS-C314759; LifeSpan Biosciences, Seattle, WA).

### Dual luciferase reporter gene assay.

Cells were cotransfected with pRL-TK and pGL4.10-*G6pc*, pGL4.10-*PEPCK*, or pGL4.10 *AC1* for 36 h. Before FSK treatment, cells were serum starved overnight. Then cells were collected 6 h after FSK treatment and assayed using the dual-luciferase reporter assay system (Promega). The renilla activity was used for normalization to account for transfection efficiency.

### Histological analysis.

The liver was excised, sectioned, and fixed overnight at 4°C in 10% formalin solution. Then the liver was dehydrated by ethanol, embedded in paraffin, and sectioned. After the dewaxing and rehydrating steps, the section was stained with hematoxylin and eosin (H&E) for histological examination. Immunohistochemistry staining was performed using a two-step detection kit (ZSGB Biotechnology, Beijing, China). Sections were incubated with primary anti-SHBs antibody (DMABT-51328MH; Creative-Diagnostics) or anti-Flag antibody (14793; CST) overnight at 4°C. Peroxidase-conjugated goat anti-rabbit antibody and substrate-chromogen were then employed to visualize the staining of the interested protein under Olympus BX53 Microscope (Olympus Corporation, Tokyo, Japan).

### Immunofluorescence staining.

For immunofluorescence staining of SHBs, the pancreatic islets were excised, sectioned, and fixed overnight at 4°C in 10% formalin solution. Antigen retrieval was conducted by boiling the slides in citrate buffer followed by gradual cooling. After being treated with 0.5% Triton X-100 for 20 min and blocked by 5% bovine serum albumin (BSA) for 30 min at room temperature, sections were incubated with anti-SHBs mouse antibody (DMABT-51328MH; Creative-Diagnostics) and anti-glucagon rabbit antibody (15954-1-AP; Proteintech Group, Inc., Wuhan, China) or anti-insulin rabbit antibody (ab181547, Abcam) overnight at 4°C. The tissue sections from AAV8-Control and AAV8-SHBs mice were then incubated with Alexa647-conjugated donkey anti-rabbit antibody (4414; CST) and Alexa594-conjugated donkey anti-mouse antibody (8890; CST). The tissue sections from LSL-SHBs and LSL-SHBs/Alb-Cre mice were incubated with Alexa488-conjugated donkey anti-rabbit antibody (4412; CST) and Alexa594-conjugated donkey anti-mouse antibody (8890; CST). Images were obtained using a confocal laser-scanning microscopy (TCS SP8; Leica, Germany).

### Statistical analyses.

Data were expressed as means ± SEM. Statistical significance was determined with a two-tailed Student’s *t* test or a one-way analysis of variance (ANOVA) followed by Tukey’s *post hoc* test. A *P* value of <0.05 was considered statistically significant.

### Ethics approval.

All experimental procedures involving the animals were performed in accordance with the National Institutes of Health Guide for the Care and Use of Animals and were approved by the Institutional Animal Care and Use Committee of Fujian Medical University.
